# Personalia

**Published:** 1914-01-17

**Authors:** 


### Personalia.

Dr. F. W. S. Stone, of Dover, has been elected chairman of the Public Health Committee.

A wedding, in which both parties were connected with the medical profession, took place in London on December 22, 1913, when Dr. W. H. Vincent, of Acton, married Miss Ruth Cracknell. The wedding presents included many from the doctor's patients, one in particular being a case of cutlery subscribed for by some of those on his panel. Both the bride and bridegroom having been attached to the staff of St. Mary's Hospital, Paddington, the wedding aroused much interest at that institution, and many presents were received from members of its staff.

The Savill Prize and Medal have been awarded to Mr. Thomas Knowles Boney, M.D., B.S. (Lond.), the subject set for the thesis being "Myasthenia Gravis." The prize and medal have been founded in memory of the late Dr. Thomas Savill, physician to the West End Hospital for Diseases of the Nervous System. Candidates are required to attend at least twenty-five demonstrations in the out-patients' department, to pass an examination, and to submit a thesis on a given subject. The next examination will probably be held in July. A wish has been expressed by the Memorial Committee that the Savill lecture should be delivered by Dr. Harry Campbell. The date will be announced later.

### FOUR COTS ENDOWED AT BLACKBURN INFIRMARY.

Mr. Lawrence Cotton, chairman of the Blackburn Rovers Football Club, has written to the secretary of the Blackburn Infirmary that to celebrate their silver wedding anniversary his brother, Mr. Clement Cotton, and Mrs. Clement Cotton desire to endow two cots in the infirmary. Mr. Lawrence Cotton adds that, though his own silver wedding is past, he and his wife also wish to endow two cots, and he encloses two cheques for £1,000 each for that purpose.

